# Evaluation of Pathology Resources for Cervical Cancer Detection Between 2018 & 2022: a Retrospective Study at Moi Teaching and Referral Hospital, Western Kenya

**DOI:** 10.21203/rs.3.rs-4791370/v1

**Published:** 2024-08-28

**Authors:** Nelson Anangwe, Jon Steimgrimson, Susan Cu-Uvin

**Affiliations:** Moi University; Brown University; Brown University

**Keywords:** Histopathology Laboratory, cervical cancer, diagnosis, LMICs, histo-technician, pathologist, Kenya

## Abstract

**Background:**

Cervical cancer cases are increasing in sub-Saharan Africa, particularly in Kenya, exacerbated by inadequate histopathology resources, posing a significant barrier to timely diagnosis and treatment. There has been little research on the availability and evolution of histopathology resources for diagnosing cervical cancer over the years. This retrospective study evaluated this evolution at Moi Teaching and Referral Hospital in Kenya between 2018 and 2022.

**Methods:**

We used a mixed-methods approach. An in-depth interview was conducted with one of MTRH’s pathology laboratory staff to assess the equipment, personnel, and quality control trends between 2018 and 2022. A thematic analysis was conducted in NVivo. We also retrospectively conducted a comprehensive inventory review of laboratory resources from 2018–2022 via purposive sampling. Microsoft Excel and Stata version 17 were utilized for descriptive statistical analysis. Turnaround time (TAT) was assessed against the UK’s National Health Service Cervical Screening Program guidelines.

**Results:**

The number of histopathology laboratory personnel at MTRH increased from 2018 to 2022, during which the facility included two pathologists, one records person, and one office administrator. Patient annual visits increased from approximately 350,000 in 2018 to approximately 500,000 in 2022. However, the histopathology personnel-to-population ratio decreased from 1.5 pathologists and 2.7 histo-technicians per 100,000 in 2018 to 1.4 pathologists and 1.8 histo-technicians per 100,000 in 2022. Despite this decrease, lab equipment, automatic tissue processors and embedding machines were added, and an average 14-day turnaround time was maintained for pathology reports.

**Conclusions:**

Despite a decreased personnel-to-patient ratio, the addition of crucial histopathology equipment mirrors the operational commitment of the Moi Teaching and Referral Hospital. The 14-day TAT is commendable, contributes to operational effectiveness and significantly contributes to timely detection. The hospital’s dedication to upgrading its infrastructure underscores a proactive approach to addressing growing healthcare demands and improving patient outcomes, even with limited human resources. The decline in the personnel-to-patient ratio underscores challenges in diagnosis, emphasizing the need to address workforce and infrastructure gaps to improve patient care within similar low-resource settings.

## Background

Cervical cancer is a chronic illness resulting from persistent infection of the cervix by HPV types 16 and 18.(Bhatla et al., 2021) In 2020, there were an estimated 604,127 annual cervical cancer cases globally, representing 3.1% of all cancer cases.(Sung et al., 2021) Approximately 84% of newly reported cases and between 87% and 90% of fatalities are concentrated in low- and middle-income countries (LMICs).(Hull et al., 2020) These cases are equivalent to more than one-quarter million deaths annually in LMICs due to inadequate human papillomavirus (HPV) vaccination programs, screening, and gross deficiencies in diagnostic infrastructure.(Cohen et al., 2019; Sung et al., 2021) In contrast, incidence rates have decreased by more than half in high-income countries (HICs) during the past 30 years due to the integration of structured screening and diagnosis initiatives.(Cohen et al., 2019) By 2030, cervical cancer mortality rates are projected to increase by approximately 25% in most LMICs. (Khozaim et al., 2014)

Histopathology has been the clinical and scientific foundation for cervical cancer diagnosis and treatment because it plays a vital role in determining the extent of the abnormality.(Bulten et al., 2011) Inadequate pathological resources for cervical cancer diagnosis can result in delayed diagnosis and treatment.(Nelson et al., 2016) As a result, the disease progresses, limiting treatment options and impeding efforts to reduce mortality. (Castle et al., 2021; Nelson et al., 2016) In sub-Saharan Africa (SSA), limited resources are crucial to integrated cervical cancer care.(Randall & Ghebre, 2016) Histopathology personnel are significantly limited in LMICs, with only approximately 30% of patients in contact with their services.(The Royal College of Pathologists, 2024) World Health Organization (WHO) reports indicate that there are approximately five pathologists per 100,000 people in HICs compared to most SSA countries, reporting approximately one pathologist per 1 million people.(WHO, 2020) For instance, the United States and Canada boast 6.5 and 4.81 pathologists per 100,000, respectively, compared to less than two pathologists per 100,000 in sub-Saharan Africa.(Metter et al., 2019; The pathologist, 2023) Due to limited pathology facilities and skilled human resources in many LMICs, the WHO’s global strategy to accelerate eliminating cervical cancer by 2030 seems to be a pipedream.(Kessler, 2017; Stelzle et al., 2021)

In Kenya, cervical cancer is the second most common type of cancer among women after breast cancer.(Kivuti-Bitok et al., 2013; Rosser et al., 2015) The quality of cervical cancer pathology infrastructure varies across regions, with rural Kenyan health facilities reporting limited access.(Ng’ang’a et al., 2018) Approximately 5,236 women in Kenya are diagnosed with cervical cancer, with an estimated 3,211 mortalities occurring annually.(ICO/IARC Information Centre on HPV and Cancer, 2021; Mwenda et al., 2022) Inadequate pathological resources are a significant barrier to timely and accurate diagnosis and treatment of cervical cancer. Timely detection and prompt initiation of treatment are critical for effectively managing cervical cancer cases.

### Specific Aims

This study assessed the progression of histopathology resources available for diagnosing cervical cancer within the Moi Teaching and Referral Hospital (MTRH) histopathology department between 2018 and 2022.

### Study Justification

The literature on cervical cancer has focused on the factors driving screening uptake and the barriers to accessing treatment in developing countries. However, there is limited research on the evolution of histopathology resources, such as the availability of skilled laboratory personnel and equipment and quality control measures across time. To address this gap, our study aimed to retrospectively evaluate resources at the MTRH histopathology laboratory between 2018 and 2022. This study’s results will contribute to the body of evidence supporting the significance of histopathology resources in reducing the burden of cervical cancer, particularly in resource-limited settings such as Kenya.

## METHODS

### Study Design and Setting

This thesis is based on primary and secondary data collected from MTRH in Western Kenya using mixed methods of qualitative and quantitative data. The facility is Kenya’s second largest national hospital, located in Eldoret town, North Rift area of Western Kenya. It has a bed capacity of approximately 1000 beds,(Odondi et al., 2020) and serves 22 counties with an approximate population of 25 million,(MTRH, 2024) representing 47% of the Kenyan population.(Jacobsen et al., 2023) We purposively selected the MTRH due to its specialized gynecological services, higher volume of cervical cancer cases, and long-standing cancer registry.

### Sampling and study population

We also purposively selected the histopathology laboratory technologist for the interview and census sampling to inventory all histopathology resources, including equipment, quality control measures, and personnel, for the specified investigation period. The histopathology department head guided the recruitment of a laboratory technologist based on their expertise, experience, and knowledge of the facility’s histopathology resources over the past ten years.

### Data collection

We informed the department of the study purpose, data utilization, and any effects of data collection on normal hospital operations. Data was collected between October 2023 and February 2024 upon pretesting of the combined histopathology inventory checklist and semi-structured interview guide. The tool was validated by a histopathology technician and a cervical cancer specialist and operationalized in English due to the interviewees’ proficiency. Upon signing an informed consent form, we interviewed the laboratory technician, corroborating the details with the histopathology inventory to capture data for the study period. We also utilized an observation checklist to document resources available in the histopathology laboratory. The guide comprised open-ended questions on three conceptual topics: 1. trained personnel were compared to the WHO’s Workload Indicators of Staffing Need (WISN) tool for laboratory staffing with a guideline ratio of more than two pathologists and two histo-technicians per 100,000 people.(Organization, 2010b) 2. Quantity and competence of *equipment*, guided by the International Standards Organization’s standards for medical laboratories, ISO 15189:2012, which recommends at least two pieces of each piece of equipment for critical function for backup purposes.(Pereira, 2020) 3. Quality control was compared to the WHO’s annual quality control performance for all histopathology laboratories, which varied by workload.(Organization, 2010a) We assessed the turnaround time (TAT) in line with the United Kingdom National Health Service Cervical Screening Program (NHSCSP) guidelines of 14 days for relaying biopsy tissue reports to patients.(Improvement, 2009)

### Data Analysis

**Figure 1:**
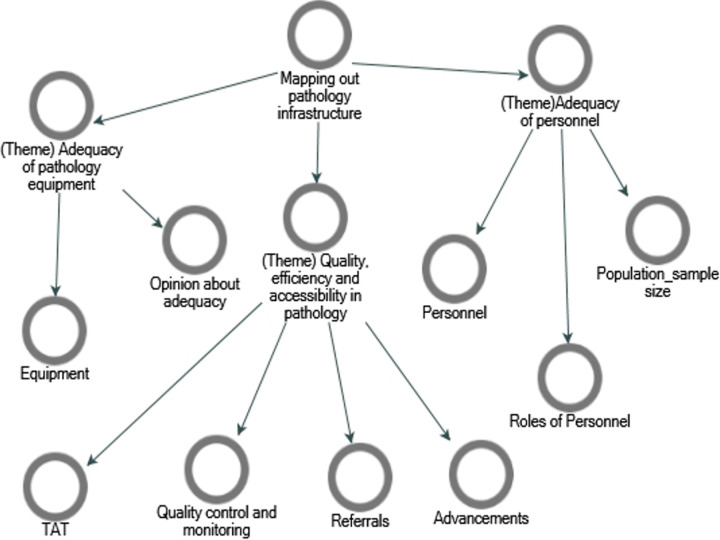
Flow diagram of emerging themes from the interviews and inventory

Initially, we thoroughly reviewed the transcript and documented observations, cross-referencing them with inventory records. Thematic analysis was performed using NVivo version 14. We then revisited the transcript reading and re-reading to familiarize ourselves with the content, noting initial codes and patterns. We employed thematic content analysis to generate critical themes.(Green & Thorogood, 2018) For this analysis, we imported *a priori* codes into NVivo and integrated them into emerging codes from the data. Following a process of open coding and identifying relevant segments, the codes were organized into more focused codes and then restructured to create descriptive codes, capturing vital concepts and relationships. This coding process enabled us to refine and revise emerging themes through an iterative process describing trends and outcomes of the findings (Fig. 1).(Strauss & Corbin, 1998)

We then merged and defined each theme grounded in the data to provide a consistent interpretation of the data. (Charmaz, 2006) Using the observational notes, we corroborated and contrasted the themes from the transcript and the inventory reports to identify patterns across the data. This enhanced the depth and reliability of your analysis.

### Ethical Approval

We sought approval from the MTRH/Moi University Institutional (no. 0004537) and Brown University Institutional Review Boards (no. STUDY00000244), after which permission was obtained from the department’s histopathology laboratory head before commencing data collection. The Data Protection Act 2019, the Health Act 2017 of Kenya, and the United States’ data privacy policy were strictly followed during the process.

## RESULTS

### Laboratory staffing

The interview highlighted key details about staffing adequacy and its impact on providing quality services across the years at the MTRH histopathology laboratory. The number of personnel at the histopathology laboratory increased: two pathologists, one records personnel, and one office administrator (Table 1). Consequently, the number of biopsy tissues examined increased from 3000 in 2020 to 7000 in 2022, with approximately 8% and 9.5% being cervical cancer biopsies, respectively.

**Figure 2:**
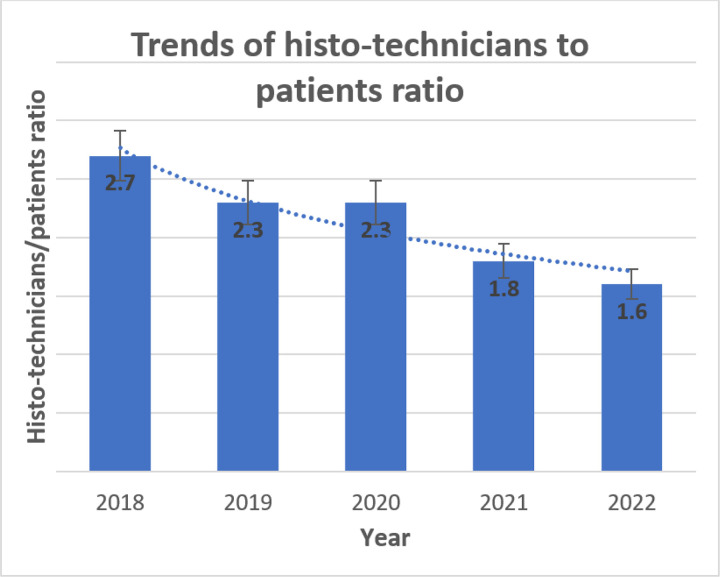
Bar graph and stacked bar chart showing trends in the histo-technicians: patient ratio

**Figure 3:**
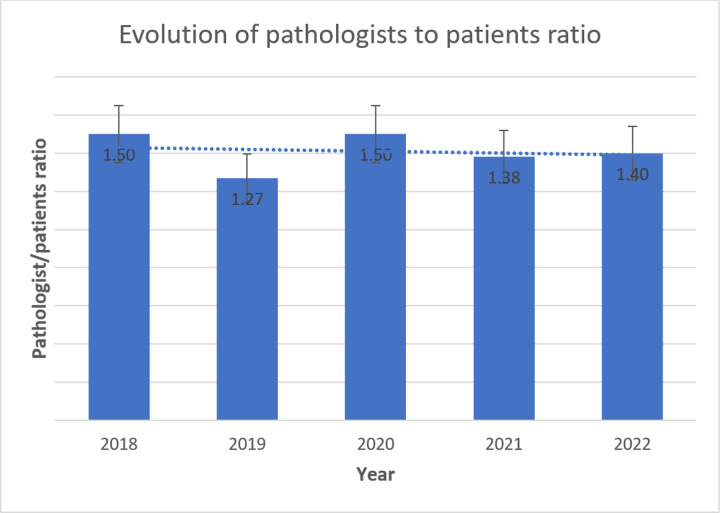
Bar graph showing the evolution of pathologist-patient ratio

The MTRH Records and Information Services Department reported providing care to approximately 350,000 and 500,000 patients annually between 2018 and 2022, respectively. Comparing the patient population to the laboratory staff, the histopathology personnel-to-population ratio decreased from 1.5 to 1.4 for pathologists (Fig. 3) and 2.7 to 1.8 for histo-technicians (Fig. 2) per 100,000 in 2018 and 2021, respectively.

### Histopathology equipment

The interview highlighted that all the equipment acquired at the commissioning of the histopathology laboratory in 2012 were manually operated. However, over the years, the facility added laboratory equipment such as an automatic microtone, automatic tissue processor, embedding machine, and immunohistochemistry machine (Table 2).

However, we noted a diminished capacity in early 2020 when a microtone machine broke down, reverting to the manual machine acquired in 2012 (Table 2). This likely impeded the efforts of biopsy tissue examination. However, the hospital eventually purchased another in 2021.

“…*The AMPATH Cervical Cancer Program donated an automatic microtome in 2018, which we used until early 2020 then it malfunctioned due to overload. The hospital eventually replaced them with two new ones in 2021*.”

The interviewee emphasized the need for automatic backup equipment for histopathology examinations, noting that manual equipment were prone to measurement errors when processing high workloads.

“*Immunohistochemistry… Yes, but we like backup machines so that in case of a breakdown, we don’t go back to manual types that are prone to many errors. So going forward, we would be comfortable when we get automatic backups*.”

### Quality control and measures

The MTRH histopathology laboratory was registered for external quality assessment (EQA) in 2018 and implemented throughout the study period (Table 3). The EQA program permits laboratories to determine the accuracy and precision of their equipment by testing samples with known characteristics such as concentration levels and other physical properties.

The MTRH Department of Biomedical Engineering evaluates all pathology equipment, personnel standards, and laboratory quality annually.

*“…an annual evaluation for all equipment in the laboratory is done by the Department of Biomedical Engineering…. Equipment servicing is performed annually by those given the contract to supply this equipment.” “…Over 80% of biopsy specimen results are obtained in 14 days.”* (Table 3)

## DISCUSSION

Our study revealed a progressive increase in pathology personnel and equipment and an increase in inpatient visits at Moi Teaching and Referral Hospital across the investigated period. These findings offer profound insights and aim to improve cervical cancer diagnostics at MTRH and hospitals in similar settings.

### Evolution of Pathology Resources at the MTRH Histopathology Laboratory

Our study included the addition of pathology personnel and equipment at the MTRH histopathology laboratory over a five-year period. In contrast to the fast-growing patient population, we noted a decrease in the laboratory staff-to-patient ratio, from 1.5 pathologists in 2018 to 1.4 pathologists per 100,000 in 2022. There was also a decrease in the histo-technician-to-patient ratio from 2.7 in 2018 to 1.8 in 2022. This finding is consistent with other studies that reported a low pathologist-to-patient ratio in SSA patients.(Khozaim et al., 2014; Metter et al., 2019; The pathologist, 2023) Limited personnel impede the performance of laboratory tasks in many SSA countries.(Khozaim et al., 2014) Abdulkareem et al.(Abdulkareem et al., 2017) reported that most countries in SSA have an average ratio of 0.1 pathologists per 100,000 people, but our study revealed a higher number of approximately 1.4 pathologists per 100,000 people in 2022.

Our results revealed a lower pathologist-patient ratio than that of most HICs, which reported approximately 6.7 pathologists per 100,000 people.(WHO, 2020) In contrast, the United States and Canada had higher histopathology-patient ratios in 2019, at 6.5 and 4.81 per 100,000, respectively.(Metter et al., 2019; The pathologist, 2023) Our findings’ numbers are lower than those from the WHO WISN guideline tool for laboratory staffing, which recommends more than two pathologists and histo-technicians per 100,000 people. This shortage likely posed a challenge in bridging the growing demand for histopathology services between 2018 and 2022. (Organization, 2010b) Research indicates that such a low staff proportion enables only 30% of patients to access urgent pathology services.(Pereira, 2020; The Royal College of Pathologists, 2024)

Despite adding an automatic microtone, an automatic tissue processor, an embedding machine, and an immunohistochemistry machine over five years, most of these malfunctioned due to overload. Our study identified a persistent challenge of relying on manual backup equipment, mainly for immunohistochemistry machines and centrifuges. Studies have shown that dependence of histopathology on manually operated equipment can lead to measurement errors, resulting in increased turnaround time and decreased efficiency in processing specimens. (Pantanowitz et al., 2009; Zardin & Braithwaite, 2018) The shortage of automated backup equipment places the MTRH histopathology laboratory below the required medical laboratories’ standards of the International Standards Organization (ISO 15189:2012) benchmark.(Pereira, 2020) These findings are consistent with a study performed in 30 sub-Saharan African countries in 2016 reporting a shortage of laboratory equipment. This study reported that immunohistochemistry machines were available in 16 countries and that molecular diagnostics were available only in two countries.(Wu et al., 2017) As reported by Thomas et al.(Randall & Ghebre, 2016), adequate pathological resources are vital in ensuring early detection of cervical cancer in the curable stage. However, according to our study, Kenya, like many low- and middle-income countries (LMICs), faces a significant scarcity of diagnostic pathology facilities and staff.(Randall & Ghebre, 2016) These shortages have often been associated with staff burnout, culminating in poor-quality services that affect the turnaround time of patient diagnostics. (Khozaim et al., 2014) These shortcomings highlight the persistent disparities in histopathology equipment inventories between LMICs and HICs.

Across the investigated period, the average turnaround time (TAT) for surgical pathology at the MTRH histopathology laboratory was maintained at 14 days. This TAT period and the implementation of annual evaluation quality control measures closely align with the UK National Health Service Cervical Screening Program (NHSCSP) guidelines of 10–14 days.(Improvement, 2009) Despite the ballooning population and increased use of cervical cancer screening services, pathology personnel’s ability to maintain a 14-day TAT despite the low ratio is worth acknowledging. This mirrors the laboratory’s effective management of its processes over time to ensure timely diagnoses despite the high number of tissue biopsies reported between 2020 and 2022. This quality measure positively contributes to operational effectiveness and patient satisfaction by reducing waiting times and anxiety.(Mumba et al., 2021)

### Strengths and limitations of the study

The strengths of this study include the use of mixed methods for retrospective data analysis to assess the histopathology laboratory inventory and the use of parallel interviews to corroborate the information for robustness. Using a five-year period of data provided a holistic understanding of the evolution of laboratory capacity, offering a nuanced and comprehensive understanding of development over time. One limitation of this study is the reliance on a single interview with laboratory staff, which may introduce interviewer bias; however, cross-referencing the pathology laboratory inventory records was essential for confirming the information and ensuring comprehensive data analysis. To enhance the robustness of future research, we recommend conducting longitudinal cohort studies that track patients over time.(Hulley et al., 2013)

## CONCLUSIONS AND RECOMMENDATIONS

Our findings highlight an improvement in the evolution of histopathology resources. However, with increasing population growth over time, the decline in the personnel-to-patient ratio underscores potential challenges in diagnosis. These shortages could further exacerbate the laboratory’s challenges in delivering timely and accurate histopathology services. This setback calls for the Kenyan Ministry of Health to allocate more funds to pathology infrastructure and personnel to improve access to essential services and reduce disparities.

A holistic approach to resource management and healthcare delivery is needed, emphasizing the need to address workforce and infrastructure gaps to improve patient care and histopathology outcomes within Moi Teaching and Referral Hospital and in similar LMIC settings.

## Figures and Tables

**Table 1:** Pathology personnel at the MTRH histopathology laboratory

	2018	2019	2020	2021	2022

Pathologists	5	5	6	7	7

Lab technologists	9	9	9	9	8

Records personnel	0	0	1	1	1

Office Administrators	1	1	2	2	2

Lab assistants	1	1	1	1	1

**MTRH patient census**	**331,036**	**393,594**	**397,399**	**507,502**	**493,780**

* The pathologist and histo-technicians to patient ratio was computed by dividing the number of the respectivepersonnel by the total number of inpatients and outpatients in each year.

**Table 2:** Inventory of Equipment

	2018	2019	2020	2021	2022

Cytology centrifuge (manual)	1	1	1	1	1

Automatic tissue processor	1	1	1	2	2

Embedding machines	1	1	1	1	2

IHC machine	1	1	1	1	1

Microtones	2	2	1	2	2

**Table 3:** Histopathology laboratory quality control

	2018	2019	2020	2021	2022

Quality checks	EQA Done	EQA Done	EQA Done	EQA Done	EQA Done

Referrals	Mostly lymphomas	Only Upon request	Only Upon request	Only Upon request	Only upon request

Turnaround time	14 days	14 days	14 days	14 days	14 days

Ref: According to the NHSCSP Guidelines, the TAT from the date the sample is taken to when the patient receives the biopsy report within 14 days

## Data Availability

The data is stored in a password protected file at the Global Health Initiative offices at Brown University (Arnold Lab 91 Waterman Street, Room 205. Providence, RI 02912) and can be requested through Nelson Anangwe, the corresponding author or Eileen Caffrey Wright, the Program Manager.

## Abbreviations

MTRH: Moi Teaching and Referral Hospital
NHSCSP: National Health Service Cervical Screening Program
WISN: Workload Indicators of Staffing Need
WHO: World Health Organization
ISO: International Standards Organization
EQA: External Quality Assessment
LMIC: Low- and Middle-Income Country
HIC: High-Income Country
TAT: Turnaround Time
SSA: Sub-Saharan Africa
HPV: Human Papilomavirus
ASCCP: American Society for Colposcopy and Cervical Pathology
KNH: Kenyatta National Hospital
AMPATH: Academic Model Providing Access to Healthcare
HIV: Human immunodeficiency virus
